# Neuroblastoma—A Review of Combination Immunotherapy

**DOI:** 10.3390/ijms25147730

**Published:** 2024-07-15

**Authors:** Barbara Pieniążek, Katarzyna Cencelewicz, Patrycja Bździuch, Łukasz Młynarczyk, Monika Lejman, Joanna Zawitkowska, Katarzyna Derwich

**Affiliations:** 1 Student Scientific Society of Department of Pediatric Hematology, Oncology and Transplantology, Medical University of Lublin, 20-093 Lublin, Poland; barbara.pieniazek56@gmail.com (B.P.); k.cencelewicz123@gmail.com (K.C.); patrycja.bzdziuch@op.pl (P.B.); 2 Department of Pediatric Oncology, Hematology and Transplantology, Poznan University of Medical Sciences, 60-572 Poznań, Poland; mlynarczyklukas@gmail.com (Ł.M.); kderwich@ump.edu.pl (K.D.); 3 Independent Laboratory of Genetic Diagnostics, Medical University of Lublin, 20-093 Lublin, Poland; monika.lejman@umlub.pl; 4 Department of Pediatric Hematology, Oncology and Transplantation, Medical University of Lublin, 20-093 Lublin, Poland

**Keywords:** cancer, neuroblastoma, combining therapy, immunotherapy, pediatric oncology

## Abstract

Neuroblastoma is the most common extracranial solid tumor found in childhood and is responsible for 15% of deaths among children with cancer. Although multimodal therapies focused on surgery, chemotherapy, radiotherapy, and stem cell transplants have favorable results in many cases, the use of conventional therapies has probably reached the limit their possibility. Almost half of the patients with neuroblastoma belong to the high-risk group. Patients in this group require a combination of several therapeutic approaches. It has been shown that various immunotherapies combined with conventional methods can work synergistically. Due to the development of such therapeutic methods, we present combinations and forms of combining immunotherapy, focusing on their mechanisms and benefits but also their limitations and potential side effects.

## 1. Introduction

Neuroblastoma (NB) is the most common extracranial solid tumor arising from the neural crest progenitor cells of the sympathetic nervous system. It is responsible for 10% of all childhood cancers and 15% of cancer deaths in the pediatric population [1]. In the United States alone, approximately 700 cases are diagnosed annually. The average age of onset for tumors is 17 months [2]. The most frequently affected areas are the adrenal glands, the abdomen, the cervical, and the thoracic paraspinal section. These solid tumors may have heterogeneous courses because parts of the tumors regress spontaneously while others are aggressive [2,3]. NB can be divided into four risk groups: very low, low, intermediate, and high risk. The classification into a given group affects the choice of treatment strategy and the patient’s prognosis [1]. If the cancer is localized, surgical treatment gives good results. However, approximately 50% of cases develop metastases, which worsens the prognosis [4]. The cure rate for low-risk and intermediate-risk NB is 90–95%, while the cure rate for high-risk neuroblastoma (HR-NB) is 50–60% [1]. The groups of patients with very low-risk and low-risk NB have the most favorable prognosis, as the 5-year overall survival (OS) in these groups reaches up to 95%. In children with medium-risk NB, this indicator is slightly lower and amounts to 80–90%. The next group consists of HR-NB patients, who constitute approximately 40% of NB cases. The prognosis in this group is the worst, as the 5-year survival rate is approximately 50–60%, which requires further research to create new therapeutic options that will improve survival in these patients [1]. NB is a clinically and biologically diverse cancer, which in turn affects its pathogenesis, prognosis, and treatment [5].

## 2. Treatment Methods in the Past and Now

In the past, all patients were treated uniformly, relying on cytotoxic chemotherapies, and most of them experienced relapses. A small population of survivors were highly burdened by the late effects of treatment. Currently, through the use of precision oncology approaches and targeted therapies, the goal is to reduce the frequency of relapses and increase the population of survivors while reducing the burden of late sequelae [6]. The management of NB depends on the risk stratification. This is presented in Table 1. For low-risk tumors, spontaneous regression is typical, so observation is in many cases sufficient. The surgical resection of the tumor achieves good results in low- and intermediate-risk NB. In addition, chemotherapy may be used before surgery to shrink the tumor or afterwards in patients with incompletely resected localized tumors or with the recurrence or progression of the disease. The treatment of HR-NB remains the greatest challenge [6,7,8,9,10,11]. The introduction of intensive multimodal treatment regimens has significantly increased patient survival [2]. The standard procedure includes: (I) the induction phase, (II) the consolidation phase, and (III) the maintenance phase, and it lasts approximately 1.5 years [12,13,14,15,16]. Prior to 2009, when treatment regimens did not include immunotherapy, less than 40% of patients with HR-NB survived 5 years or more without relapse [12]. The implementation of immunotherapy was one of the factors that increased the OS of HR-NB patients from 29% to 50% for patients diagnosed from 1990 to 1994 and from 2005 to 2010, respectively [14].

The intensive multimodality treatment of HR-NB is associated with long-term consequences. Survivors have been reported to suffer from treatment-related endocrinopathies, hearing loss, growth failure, musculoskeletal abnormalities, cardiopulmonary sequelae, renal toxicities, impaired gonadal function, infertility, and second malignant neoplasms (e.g., myelodysplasia and leukemia), among others [17,18,19,20]. With the improved survival rate of patients with HR-NB, there is an increasing need to develop new strategies that will reduce the risk of toxicity and long-term consequences and enhance patients’ quality of life. New immunotherapeutic strategies bring hope.

## 3. Tumor Biomarkers

An important aspect regarding the treatment of NB, which enables the adaptation of the appropriate treatment regimen to the patient and the assessment of the prognosis, still remains the presence of tumor biomarkers. They are specific indicators of the biological state of a specific tumor, providing information on the evolution of the disease. Since therapeutic strategies in NB are still not gratifyingly effective, a better understanding of the biomarkers of this cancer seems to be an important part of making diagnosis and treatment methods more effective [21].

The most significant of the genetic biomarkers of NB known so far remains the amplification of the *MYCN* gene, which regulates the metabolic pathways of the tumor, and its levels correlate with the course of the disease. It is effective in detecting recurrence at an early stage and allows for the monitoring of disease progression (PD) [21,22]. Also, the anaplastic lymphoma kinase (*ALK*) gene has an important prognostic function in NB; it has been proven to play a role in the genesis of this disease. As it turns out, the *ALK* mutation correlates with a medium and high risk of NB and low survival rates [21].

As in other cancers, serum protein levels—although not very specific as they also increase in noncancerous diseases—can be used as NB biomarkers [22]. The level of lactate dehydrogenase (LDH) turns out to be an important prognostic biomarker. Its increase correlates with a severe course of NB, a high risk of metastasis, recurrence, and a bad prognosis. However, its diagnostic significance is presumed to work only in patients without *MYCN* amplification [21]. An unfavorable prognostic biomarker at the time of diagnosis is an increase in serum glycosylated ferritin. It is observed to increase markedly in stages 3 and 4 of the disease [22]. Urinary catecholamine levels are also of clinical value in assessing the prognosis of NB. It has been observed that a low vanillylmandelic acid (VMA)-to-homovanillic acid (HVA) ratio is associated with an unfavorable prognosis in patients. An analysis of both VMA and HVA has been shown to have high diagnostic sensitivity, and it is, therefore, a good tool for predicting the course of this disease [21,22].

However, as therapeutic models based on combination immunotherapy are relatively new, tumor biomarkers that will provide information on the response to and success of such treatment are still being sought after.

**Figure 1:**
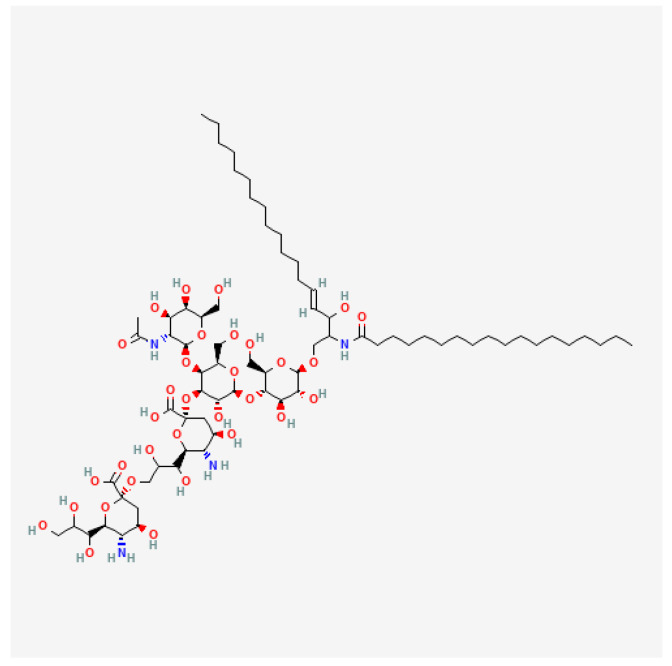
Two-dimensional Chemical Structure of GD2 Ganglioside [27].

Currently, GD2 seems to be of the greatest importance in this regard. GD2, whose chemical structure is presented in Figure 1, is a glycolipid antigen—ganglioside which belongs to the glycosphingolipids containing sialic acid in their structure. The functions of these compounds include primarily signal transmission and participation in cell recognition and adhesion. GD2 is built from a hydrophobic and hydrophilic part. Ceramide, as the hydrophobic part, enables GD2 to bind to the cell surface by interacting with membrane lipids, and the hydrophilic head enables interactions with other membrane molecules or extracellular molecules [23,24,25].

High levels of GD2 correlate with an advanced disease stage, rapid progression, and low survival rates. It has been tentatively shown to be a useful diagnostic and prognostic biomarker and—which is equally important—highly specific for the disease NB. However, more studies are needed to confirm the clinical value of GD2 as a biomarker for NB. Additionally, a further search for biomarkers is needed to make therapy more effective [26].

## 4. Combining Immunotherapies Based on Dinutuximab

### 4.1. Characteristics of Dinutuximab and Dinutuximab Beta

**Figure 2:**
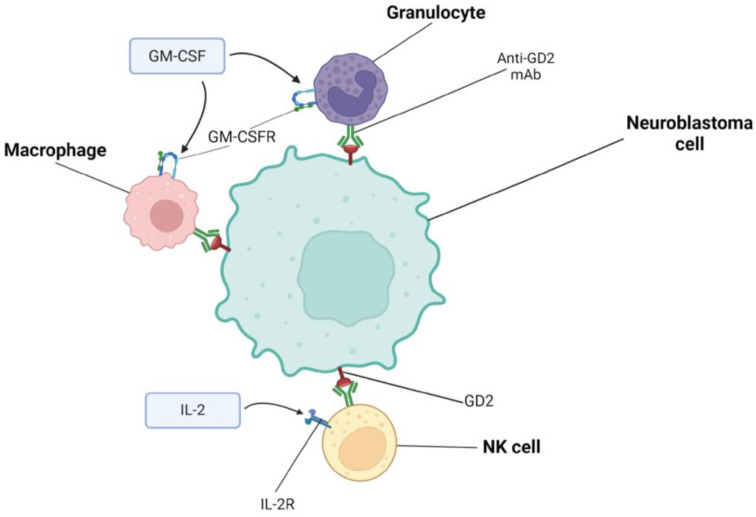
Mechanism of action of anti-GD2 monoclonal antibody: anti-GD2 mAb binds to GD2 disialoganglioside, which is highly expressed on the surface of NB cells and activates complement. Anti-GD2 monoclonal antibody mediates CDC and ADCC, which are induced by neutrophils and NK cells. These cells bind antibodies. ADCC and, thus, the antitumor effect can be increased using immunostimulating cytokines: IL-2 targeting the interleukin-2 receptor (IL-2R) and GM-CSF targeting the GM-CSF receptor (GM-CSFR). Created with BioRender.com (accessed on 24 June 2024).

GD2 is highly expressed on the surface of NB cells, and in healthy tissues, it is weakly expressed on neurons, melanocytes, and peripheral pain fibers. Importantly, GD2 present on NB cells is immunomodulated only at the cell membrane and circulating levels in cerebrospinal fluid and blood are not sufficient to impede its binding by specific antibodies [28]. This makes GD2 an attractive target for NB-specific immunotherapy. It is worth noting that the presence of GD2 disialoganglioside on the pain fibers of peripheral nerves is also associated with side effects. Dinutuximab is an IgG1 monoclonal antibody directed against GD2 found on the surface of NB cells [29]. Hence, in 2015, on 10 March, the Food and Drug Administration approved it for the treatment of HR-NB. Dinutuximab mediates complement-dependent cytotoxicity (CDC) and antibody-dependent cytotoxicity (ADCC). Dinutuximab coats NB cells but also binds via its Fc portion to FcγRIIIA and/or FcγRIIC, which are mainly found on NK cells but are also present on macrophages and some circulating monocytes. These events induce ADCC, which relies mainly on the release of perforins and gransins to lyse NB cells. Moreover, the production of IFNγ is also triggered and NK cells may begin to transmit death signals to NB cells. In turn, the stimulation of FcgRIIa and FcgRIIIa receptors on the surface of macrophages activates the production of cytotoxic proteases, free oxygen radicals, and cytokines, and also activates the process of the phagocytosis of cancer cells coated with antibodies. Dinutuximab may also participate in CDC because the Fc fragment of the antibody contains sections responsible for complement activation. Dinutuximab binds to the complement component C1q, which activates the complement cascade and formation of the membrane attack complex (MAC), leading to the lysis of NB cells. Studies have shown that complement activation also has other effects, affecting innate and adaptive immunity. This response is mediated by soluble complement fragments (C3a and C5a) and immune cells (NK cells, neutrophils, macrophages, T lymphocytes, and dendritic cells), whose complement receptors are stimulated [30,31,32,33,34,35]. In order to increase ADCC and, thus, the antitumor effect, immunostimulatory cytokines can be used such as interleukin-2 (IL-2) and granulocyte-macrophage colony-stimulating factor (GM-CSF), as presented in Figure 2. However, the study results showed that adding IL-2 to maintenance immunotherapy does not bring clinical benefits and only increases the toxicity of the therapy. The probable cause of such results is the masking of the positive effect of IL-2 due to the preferential expansion of regulatory T cells (Tregs) [36]. Therefore, it was suggested that a new anti-GD2 combination with more specific methods of immune enhancement should be sought. Isotretinoin is also often administered in dinutuximab therapy [34].

Dinutuximab beta (DB) is a chimeric human–mouse IgG1 monoclonal antibody (mAb) directed against disialoganglioside GD2. DB is produced in the mammalian Chinese hamster ovary (CHO) cell line using recombinant deoxyribonucleic acid (DNA) technology. This drug was approved by the European Medicines Agency (EMA) in 2017 for the treatment of HR-NB in patients from 12 months of age who achieved at least partial response (PR) on induction chemotherapy and received myeloablative therapy and stem cell transplantation and in patients with a history of relapsed/refractory (R/R) HR-NB. DB is used with 13-cis-retinoic acid (RA) for maintenance therapy and may be combined with IL-2 in patients with a history of R/R disease, as well as in patients who have not achieved a complete response (CR) to first-line therapy. However, studies have been conducted that did not show any benefit of combining IL-2 with DB in HR-NB and recurrent NB but showed significant toxicity. GM-CSF increases anti-GD2 activity, but it is not available in Europe [37].

### 4.2. Dinutuximab and Irinotecan, Temozolomide, GM-CSF

**Figure 3:**
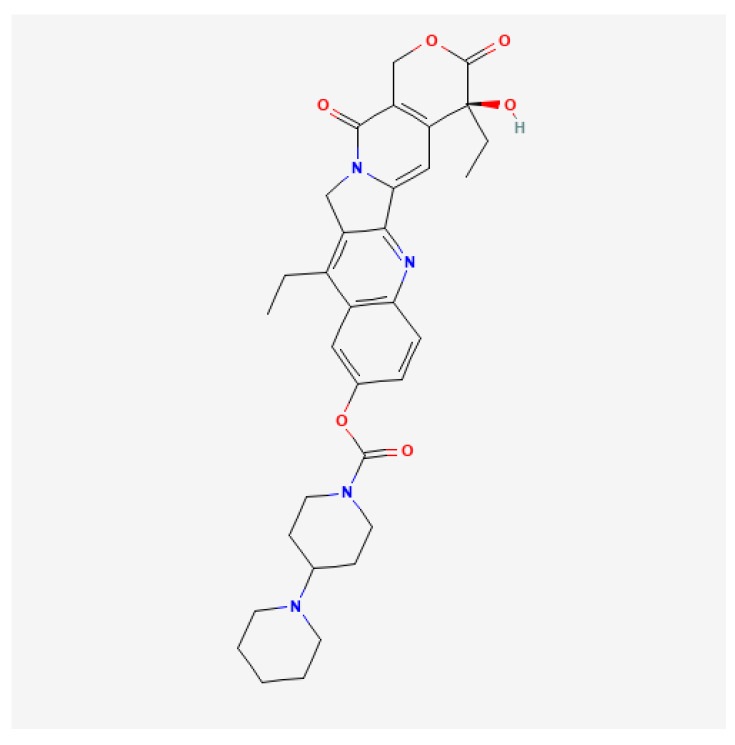
Two-dimensional Chemical Structure of Irinotecan [39].

**Figure 4:**
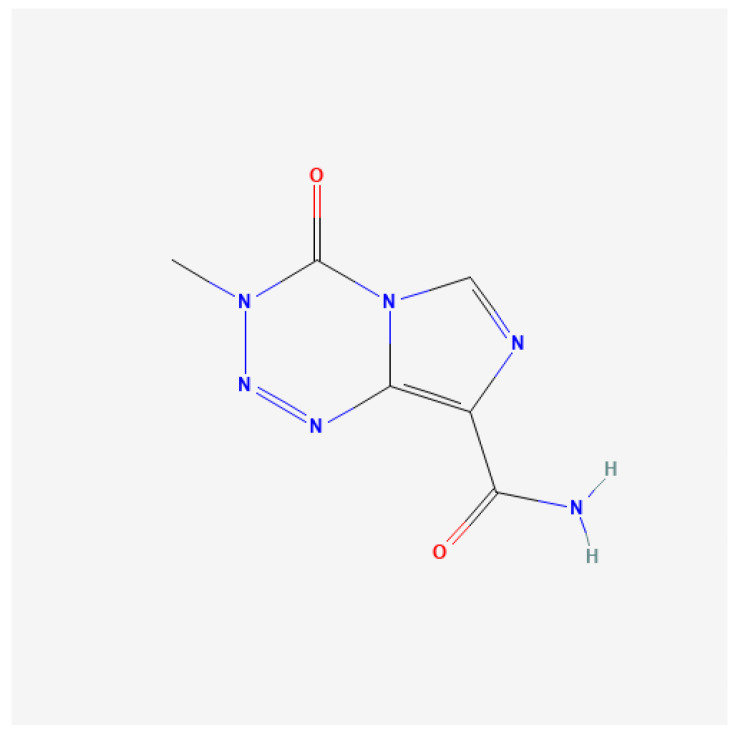
Two-dimensional Chemical Structure of Temozolomide [40].

Clinical studies. Mody et al. conducted a randomized trial ANBL 1221 consisting of two stages. In the first stage, which lasted from February 2013 to March 2015, 17 patients qualified and were randomly assigned to irinotecan and temozolomide—whose chemical structures are presented in Figure 3 and Figure 4—dinutuximab, and GM-CSF (I/T/DIN/GM-CSF). In the second stage, lasting from August 2016 to May 2017, 36 additional patients were enrolled and were non-randomly assigned to I/T/DIN/GM-CSF therapy. Non-random assignment occurred through the National Cancer Institute’s OPEN system. Patients with R/R NB were included in the study and their age at enrollment ranged from 1.3 to 15.9 years. Regarding *MYCN* status, it was known among 51 patients; 14 of them had tumors with *MYCN* amplification. At the time of inclusion in the study, 37 people had a measurable disease (69.8%), and in 16 patients, the disease was evaluable (30.2%). In total, 31 people were diagnosed with a treatment-resistant disease (58.5%) and 22 people had a recurrence of NB. The best overall response (OR) was observed in 9 of 17 patients in the first part of the study (52.9%) and in 13 of 36 patients enrolled in the second part of the study (36.1%). Most responders achieved the best OR at the first time of disease evaluation, and approximately 40% achieved this response at cycle ≥ 4. No patients achieved an OR for the first time after cycle 6. In summary, 22 of 53 patients had an OR, of which 11 achieved a CR and 11 achieved a PR. Twenty-two patients had stable disease (SD), and PD was observed in seven patients. Two people were not assessed. In patients whose disease showed progression, the sites of progression included bone (one person), metastatic soft tissue (two people), primary site (one person), and combined sites (two people). The one-year progression-free survival (PFS) value for all patients was 67.9% ± 6.4% (95% CI), and the median time to event was 0.8 years. The one-year OS was 84.9% ± 4.9% (95% CI), and the median time to death was 1.1 years. Adverse events were observed during the study and are presented in Table 2. Hypoxia and peripheral motor neuropathy were also reported in the randomized cohort. In the expanded cohort, two patients experienced grade 3 hypoxia; none experienced motor neuropathy. Twelve patients required I/T dose modification, and DIN dose modification was necessary in fourteen patients. In the initial cohort, patients who experienced hypoxia and bronchospasm had their treatment discontinued due to toxicity [38].

Lerman et al. conducted a retrospective study that included 146 patients. The study patients were treated at one of 13 children’s hospitals, and relevant demographic and clinical data were recorded in the secure REDCap database. The study included people who were diagnosed with HR-NB before the age of 30 and received ≥1 cycle of I/T/DIN/GM-CSF due to disease progression or recurrence. The median age at diagnosis of HR-NB was 51 months. Patients with primary refractory disease who progressed via induction were excluded from the study. The response to the implemented treatment was assessed using the International Neuroblastoma Response Criteria. Among 134 patients whose tumor *MYCN* status was known, *MYCN* was amplified in 43 (32%) of them. A total of 94 patients had a known tumor *ALK* status and 21 (22%) of them had tumors with gene amplification or activating mutation. ALK is a receptor tyrosine kinase involved in cell proliferation and neuronal differentiation. Activating an *ALK* mutation stimulates excessive cell proliferation and abnormal differentiation in NB tumors; therefore, targeting the ALK receptor with ALK inhibitors also has potential in NB therapy [1,41,42]. I/T/DIN/GM-CSF included the treatment of the first relapse or progression in 99 (68%) patients; 92 (63%) patients had measurable disease at recurrence or progression; and before I/T/DIN/GM-CSF therapy 81 (56%) patients had received anti-GD2 therapy. During I/T/DIN/GM-CSF therapy, 34 (23%) patients were treated at some point with surgery (n = 5), radiotherapy (n = 23), metaiodobenzylguanidine (mIBG) (n = 2), or other anticancer therapy (n = 5). I/T/DIN/GM-CSF was discontinued in 85 (59%) patients due to insufficient response—SD or PD—in 19 (13%) due to unacceptable acute/chronic toxicity and in 29 (20%) patients due to the completion of treatment planned by the doctor and/or family. Among 146 patients, 71 (49%) had an OR after I/T/DIN/GM-CSF therapy (CR—29%, PR—15%, and minor response (MR)—5%); 31 (21%) patients achieved SD; and in 44 (30%) patients, the disease progressed. In patients who initially presented with SD, MR, or PR, the best response was achieved in 57 of 79 (72%) at the first disease evaluation. Only one patient needed >6 cycles to achieve the first OR. A total of 108 patients experienced disease recurrence or progression within 0–30 months of starting I/T/DIN/GM-CSF treatment. The most common sites of recurrence or PD were bone (77%), soft tissue and lymph node metastases (55%), bone marrow (31%), the lungs (17%), and the brain (6%). The median PFS among all patients receiving I/T/DIN/GM-CSF was 13.1 months (95% CI); the 6-month PFS was 62% (95% CI), the 1-year PFS was 51% (95% CI), and the 2-year PFS was 28% (95% CI) [42].

This retrospective study confirmed that the response rates to chemoimmunotherapy—I/T/DIN/GM-CSF administered for disease relapse observed in study ANBL1221—are reproducible in clinical practice outside the study context, and the initial time to response to I/T/DIN/GM-CSF varies [38,42].

### 4.3. Dinutuximab and Tipifarnib

Tipifarnib (R115777) is the first selective farnesyltransferase inhibitor (FTI) to enter clinical trials [43]. However, it has not been approved by the FDA for any clinical indication based on suboptimal Phase III trial results due to its toxicity. Typical toxic effects occurring after its use include neurotoxicity and bone marrow suppression. Despite the FDA’s rejection of tipifarnib, it is still under investigation for its treatment of acute myeloid leukemia [44,45]. Tipifarnib is a heterocyclic non-peptidomimetic drug that inhibits the farnelization of the canonical lamin FTase substrate with subnanomolar potency. Farnesyl transferase is required for the secretion of small extracellular vesicles (sEVs), which can also originate from NB cells, and their functions include modulating the tumor environment (TME) and the host immune system. Tumor-derived sEVs have been shown to cause the dysregulation of NK cell function and depletion. As is known, NK cells are the main effector cells in anti-GD2 immunotherapy (including dinutuximab) and use ADCC to target NB cells. This is also supported by studies showing that the presence of NK in the tumor mass is a strong positive prognostic factor in several types of solid tumors, including NB. With these data, tipifarnib may continue to be considered for the treatment of NB, as preclinical studies have shown that it inhibits the release of sEVs that increase resistance to dinutuximab. Thanks to FTI, the inhibition of sEV secretion may increase the effectiveness of dinutuximab and prevent the development of sEV-induced immunosuppression [43,45,46].

Preclinical studies. Liu et al. conducted a study using the murine NB cell line 9464D and the human NB cell lines IMR32 (CCL-127), HEK 293T/17 (CRL-11268), and NK92-EGFP-CD16 (PTA-8836). An immunocompetent mouse model of NB was generated with male and female mice represented in equal proportions. One week after tumor cell implantation, mice were randomly assigned to treatment groups. According to random assignment, the group received phosphate-buffered saline (PBS), dinutuximab, purified sEV from 9464D-GD2 cells, or a combination of dinutuximab and sEV. Where indicated, tipifarnib or an equivalent volume of vehicle was administered. Tumor volume was calculated by measuring the tumors using calipers. NB cells were also divided into groups. Some cells were incubated with NB-derived sEVs before dinutuximab was added to the total. An Incucyte S3 live cell imaging system (Sartorius) was used and quantified using the Incucyte cell-by-cell analysis software module (Sartorius). The study showed that NB-derived sEVs significantly attenuated the effectiveness of dinutuximab in vivo and modulated the infiltration of tumor immune cells after anti-GD2 antibody treatment, thereby causing an immunosuppressive effect on the TME, which contains more tumor-associated macrophages and fewer NK cells infiltrating the tumor. Furthermore, NB-derived sEVs inhibit splenic NK cell maturation in vivo and dinutuximab-induced ADCC in vitro. Tipifarnib was shown to significantly enhance dinutuximab-mediated tumor growth inhibition and prevent the immunosuppressive effects of NB-derived sEVs in vivo. In summary, the tipifarnib-mediated inhibition of sEV secretion may serve as a viable treatment strategy in the future to enhance the antitumor efficacy of anti-GD2 immunotherapy in patients with HR-NB [45].

### 4.4. Dinutuximab Beta and Irinotecan, Temozolomide

Clinical studies. Olgun et al. conducted a study using DB, irinotecan, and temozolomide (IT). This study lasted from January 2020 to March 2022. Patients older than 12 months with a documented diagnosis of HR-NB in the event of relapse or refractory treatment were enrolled in the study. Other inclusion criteria included the disease being measurable using contrast-enhanced magnetic resonance imaging (MRI) and/or computed tomography (CT) or mIBG/fluorodeoxyglucose (FDG) positron emission tomography (PET)/CT and/or detected based on bone marrow aspiration and biopsy. After the diagnosis, all patients received treatment according to the 2009 national NB protocol of the Turkish Pediatric Oncology Group. At the time of relapse or progression, rescue treatment was initiated. The drug combinations used included ICE (ifosfamide + carboplatin + etoposide), TVD (topotecan + vincristine + doxorubicin) or TVC (topotecan + vincristine + cyclophosphamide) ± temsirolimus ± bevacizumab), RIST (rapamycin, irinotecan, sunitinib, temozolomide), and IT. The combination of IT was the most popular. Patients whose only localization of the disease was bone marrow were not eligible for the study. The study included 19 patients who received a total of 125 cycles of DB + chemotherapy. The median age at the time of inclusion in the study was 5.5 years. All patients then had INRG stage M disease, and one patient had central nervous system disease. *NMYC* amplification was detected in 3 patients, 14 patients had no *NMYC* amplification, and 2 patients had an unknown *NMYC* amplification status. At the time of enrollment in the study, all patients except one had bone metastases. Bone marrow metastases were present in 13 patients; in 3 patients, the dura mater was involved; and in 8 patients, a soft tissue tumor was detected. At baseline, 10 patients had a relapsed disease and 9 had a refractory disease, 2 of which had PD. OR was achieved in 12 (63%) patients, including 6 CR patients and 6 PR patients. No disease was detected in bone marrow biopsies in any of the patients who achieved an objective response. Patients with a CR achieved their best response after six cycles, and patients with a PR received three to seven cycles of DB and chemotherapy. SD was observed in two patients and PD in five patients. Four patients died of PD, with one achieving bone marrow CR after the fifth cycle but developing PD after the ninth treatment cycle. Adverse events were graded according to the NCI Common Terminology Criteria for Adverse Events (CTCAE). The most common adverse reactions are listed in Table 2, including grade ≥3 adverse reactions. Anaphylaxis was not observed in any patient. Rare side effects include mild hypoxia (≥90% O_2_) without other accompanying symptoms. One patient experienced swelling of the cheek and face during almost every treatment, but this did not result in the discontinuation of treatment. In summary, immunotherapy-induced toxicity was transient, and the use of appropriate supportive therapy or the interruption of antibody infusion was sufficient to resolve the toxicity. The results of this study demonstrate that DB-based chemoimmunotherapy is safe and effective in patients with R/R HR-NB [37].

Wieczorek et al. performed a retrospective review of the clinical charts of patients with R/R HR-NB who received treatment with a combination of chemotherapy and immunotherapy with DB on a compassionate-use basis. Twenty-four patients received chemotherapy with irinotecan in combination with temozolomide (TEMIRI), and one patient received topotecan because PD was diagnosed while previously receiving TEMIRI with bevacizumab. Patients were treated at the Krakow center or the center in Greifswald, Germany, from December 2017 to October 2021. Patients were classified as HR-NB patients based on the International Neuroblastoma Staging System (INSS). These were patients aged 12 months or more and had NB stage 4 INSS or stages 2–4 or 4S INSS with *MYCN* amplification. Additionally, the disease was required to be measurable/assessable. Patients with a disseminated recurrence were also included in the study. A total of 25 patients were included in the study. *MYCN* amplification was found in 11 (44%) patients, and unfavorable histology was found in 16 (64%) patients. In total, 72% of patients received first-line treatment according to the HR-NBL SIOPEN protocol, and 14 (56%) of patients received first-line maintenance treatment with DB. Among the 25 patients enrolled in the study, 20 (80%) received immunotherapy combined with chemotherapy for relapsed disease, while the remaining 5 (20%) received this treatment for refractory disease. Patient response to therapy was assessed using the International Neuroblastoma Response Criteria. The following results were achieved when DB was administered in combination with irinotecan/ temozolomide. CR and PR were achieved in 8 (32%) patients, resulting in the best objective response rate (ORR) of 64% (16/25). Of the 16 patients who achieved the best response, 11 (69%) achieved the best response after a maximum of 5 treatment cycles and 5 (31%) achieved the best response after 6–8 treatment cycles. SD was noted in five patients and no response (NR) was observed in four patients, confirming PD. The median OS from the start of chemoimmunotherapy was 10.3 months, and the median PFS was 6.3 months. The OS rate was 47% after one year and 35% after three years, and the PFS after one year was 48% and after three years it was 36%. During the treatment, side effects were observed and are presented in Table 2. Late treatment complications were also observed. Hypothyroidism occurred in four patients, chronic kidney disease occurred in one patient, and focal nodular hyperplasia of the liver occurred in another (this was not related to disease recurrence). One patient had leukopenia and neutropenia, but they were not related to bone marrow dysfunction. Toxicity was comparable to those previously reported for individual therapies and was not severe enough to result in a decision to discontinue treatment due to toxicity [47].

**Table 2:** Toxicity profile of therapies based on dinutuximab, dinutuximab beta, and naxitamab.

Antibody	Molecular Target	Combined Therapy Model	Common Toxicities	Uncommon Toxicities	ClinicalTrials.gov Identifier	References
Dinuxitimab	GD2	Dinutuximab, irinotecan, temozolomide, GM-CSF	Fever Neutropenia Pain Diarrhea	Vomiting Thrombocytopenia	NCT01767194	[38,48]
Dinuxitimab beta	GD2	Dinutuximab beta, irinotecan, temozolomide	Leukopenia Neutropenia Fever Anemia Thrombocytopenia Hypertransaminemia Diarrhea	Tachycardia Vomiting Pain Anorexia Rash and itching Hypertension Capillary leak syndrome Allergy	NCT05272371	[37,47,49]
Dinuxitimab beta	GD2	Dinutuximab beta, haplo-SCT	Hypertransaminemia Anemia Proteinuria Fever Constipation Diarrhea Pain Skin toxicity	Leukopenia Neutropenia Thrombocytopenia Creatinine elevation Capillary leak syndrome Stomatitis Bilirubin elevation Allergy Nausea Vomiting Neurotoxicity Hypotension	NCT02258815	[50,51,52]
Naxitamab	GD2	Naxitamab, irinotecan, temozolomide, GM-CSF	Anemia Neutropenia Thrombocytopenia Hypotension Pain Hypertension Diarrhea	Bronchospasm Anorexia Urticaria Skin ulceration Vomiting Swelling of the larynx Postural disease Hypertransaminemia Neutropenic fever	-	[53]

### 4.5. Dinutuximab Beta and Haplo-SCT

DB, which is an anti-GD2 antibody, presented in Table 3, as mentioned above, acts through ADCC and CDC. Because cytotoxic therapies used to treat NB may impair the ability of NK cells to mediate ADCC, it is important to restore their functional population. This can be executed by transplanting stem cells from haploidentical familial donors (haplo-SCT). Furthermore, studies have shown that DB improves outcomes after autologous stem cell transplant (ASCT) during first-line treatment. Therefore, it seems essential to administer DB in combination with haplo-SCT in order to enhance the early expansion and durability of NK cells that will come from the donor [50,51].

Clinical studies. Flaadt et al. retrospectively reviewed the data of 13 patients with HR-NB who had a central nervous system (CNS) relapse and who received multimodal therapy with consolidating haplo-SCT followed by DB plus subcutaneous interleukin-2 (scIL-2). People who underwent the above-mentioned therapies between September 2010 and October 2021 were included in the study. Most patients (11/13, 85%) had stage 4 NB at diagnosis; one patient initially classified as stage 4S was reclassified as stage 4; and one patient had stage 3 NB with *MYCN* amplification. Four (31%) eligible patients were previously treated after DB consolidation. DB was started 60–180 days after transplantation if the patient had no symptoms of graft-versus-host disease (GvHD) and did not require immunosuppressive therapy. Three patients were not included in the feasibility study; they underwent haplo-SCT but only received five cycles of DB. ScIL-2 was not administered to these patients. Following treatment, CR was achieved in 9 of 13 patients, while 4 patients died (2 due to PD and 2 due to serious adverse events unrelated to DB treatment). Patients who were alive (as of July 2023) remained disease-free. The 5-year event-free survival (EFS) rate from the start of relapse treatment was 55.9% (95% CI), and the 5-year OS rate was 65.3% (95% CI). Grade 3/4 hematologic adverse events occurred in eight patients. Among the grade 3/4 non-hematological adverse events, the most common were those affecting the patient’s general condition and are listed in Table 2. One patient’s DB treatment was discontinued after cycle 7 due to a serious adverse event (chronic hemolysis). The therapy was generally well tolerated and any side effects were manageable. Despite the study’s limitations, it demonstrated that multimodal therapy with consolidating DB after haplo-SCT (with or without ScIL-2) appears to be a promising therapeutic option for patients with recurrent HR-NB [50].

Flaadt et al. conducted a prospective, single-arm, open-label, phase I/II study. Seventy patients from four European centers were screened from November 2010 to November 2017; two patients failed the screening. Ultimately, 68 patients were included and analyzed. The median age upon study entry was 6.5 years (range of 3–20); all but four patients (94.1%) had metastatic disease at the time of recurrence. The eligibility criteria were their age at enrollment (1–21 years), having R/R NB stage 4 according to the INSS or recurrent NB stage 2–3 with *MYCN* amplification, and having haplo-SCT as part of the relapse treatment study. *MYCN* amplification was present in 19 (29%) patients, 46 (71%) patients had no *MYCN* amplification, and 3 patients had unknown *MYCN* amplification status. The transplant was performed on patients according to the guidelines. During the transplant, the patients received ex vivo peripheral stem cells depleted of T and B lymphocytes after myeloablative conditioning. Then, from day 6 after the transplantation, patients who did not experience GvHD or acute GvHD ≤ grade 2 could be administered DB. Lower DB doses and longer infusion rates were consistent with study recommendations and were due to hypersensitivity reactions. To avoid GvHD induction, a low dose of scIL-2 was administered only in selected cycles and on selected days. A total of 62 (91.2%) patients received scIL-2 as recommended; in 5 (7.3%) patients, the method of administration was unknown. One patient (1.5%) did not receive scIL-2. The occurrence of GvHD delayed DB administration, resulting in a median time to start DB administration after haplo-SCT for all patients of 91 days (range of 61–363 days). Ten (14.7%) patients received anti-GD2 therapy during first-line or relapse treatment. Before haplo-SCT, 16 (24%) patients achieved CR; 39 (59%) patients showed PR; and 11 (17%) showed NR, mixed response, or PD. Residual disease was present in 50 patients before haplo-SCT. After haplo-SCT and before the first cycle of DB, 25 (36.8%) patients were in CR, 35 (51.5%) patients were in PR, and 8 (11.8%) were NR/MR/PD. The ORR in 43 patients with evidence of disease after haplo-SCT was 51.2%, with a CR rate of 34.9%. Some patients (n = 29; 42.6%) did not complete the trial treatment due to PD, therapy-related toxicity (hypersensitivity/inflammatory reactions), hemolytic anemia, posterior reversible encephalopathy syndrome (PRES)/CNS toxicity, infections (human herpes virus 6 and bacterial sepsis), or patients’ decisions to discontinue immunotherapy. In summary, during the study, 39 (57.4%) patients completed six cycles, 13 (19.1%) patients maintained CR, 15 (22.1%) achieved CR, 6 (8.8%) patients had PR, 2 (2.9%) of the patients had SD, and 3 (4.4%) had PD at the end of treatment. Relapse/progression occurred in 34 patients at a median time of 235 days. At the last follow-up in October 2021, 35 (51.5%) patients were alive. The causes of patient death included disease recurrence, infections, PRES, and secondary malignancy. The 5-year EFS rate from the trial’s initiation in the entire cohort was 53% (95% CI), and the 5-year OS was 43% (95% CI). Toxicity was recorded according to CTCAE4.0. Grade 3/4 hematologic adverse events occurred in 29 (42.6%) and are listed with the most common grade 3/4 nonhematologic adverse events in Table 2. In addition, viral, fungal, or bacterial infections were observed [51].

**Table 3:** Antibodies approved for NB treatment.

Antibody	Clonality	Type	Molecular Target	Approval for Treating NB	Trade Name	References
Naxitamab	monoclonal	humanized	GD2	2020	DANYELZA	[54]
Dinuxitimab	monoclonal	chimeric	GD2	2015	UNITUXIN	[55]
Dinuxitimab beta	monoclonal	chimeric	GD2	2017	QARZIBA	[56,57]

## 5. Combining Immunotherapies Based on Naxitamab

### 5.1. Characteristics of Naxitamab

Naxitamab is an anti-GD2 mAb used to treat GD2-positive cancers. GD2 is a disialoganglioside that is overexpressed in neuroectodermal-derived cells [54]. In 2020, naxitamab was approved for the treatment of NB in adults and in at least one-year-old children with R/R forms of the disease [58]. Immunotherapy targeting GD2 is becoming a part of the core protocols for treating patients who have not experienced remission despite first-line therapy. The potential for enhancing the effect of anti-GD2 antibodies with chemotherapy has been discovered. Two primary treatment schemes based on naxitamab are naxitamab with GM-CSF and naxitamab with irinotecan, temozolomide, and GM-CSF [38,59,60]

### 5.2. Naxitamab and Irinotecan, Temozolomide, GM-CSF

Clinical studies. Muñoz et al. conducted a retrospective study that evaluated the efficacy and toxicity of naxitamab with irinotecan, temozolomide, and GM-CSF therapy and survival among patients with HR-NB refractory to current treatment. Chemoimmunotherapy cycles included irinotecan at 50 mg/m^2^/day intravenously; temozolomide at 150 mg/m^2^/day per os on days 1–5; naxitamab at 2.25 mg/kg/day intravenously on days 2, 4, 9, and 11; and GM-CSF at 250 mg/m^2^/day subcutaneously on days 6–10. Cycles were administered in an outpatient setting every 4 weeks. There were two cohorts that included 17 patients each—cohort 1, which received early chemoimmunotherapy (median 8.4 months from diagnosis to treatment), and cohort 2, which received late (post-induction) chemoimmunotherapy (median 1.4 years from diagnosis to treatment). The results were as follows: in cohort 1, the best results at any moment were CR = 47% and SD = 53%. Relapse occurred in three of eight patients with CR and progression occurred in three of nine patients with SD. After the follow-up period, 14 (82%) patients survived and 3 patients died from the disease. The 3-year OS was 84.8% (CI = 67.4%). In cohort 2, the best response was CR = 12%, PR = 6%, SD = 53%, and PD = 29%. None of the patients with CR had disease relapse while seven of eight patients who received disease stabilization ultimately progressed. After the follow-up period, 6 (35%) patients survived and 11 patients died from the disease. The treatment of one patient was discontinued due to toxicity. The 3-year OS was 29.4% (CI = 12.8%). During the study, 29 of 34 patients developed adverse reactions, which are presented in Table 2. One patient developed grade 4 anaphylaxis on the first day, so his treatment was stopped. Considering that none of the patients developed grade 5 toxicity, and grade 4 toxicity occurred in only one patient, the treatment can be considered relatively safe. The results of the study strongly suggest that refractory disease can be overcome using this method, but it is important to apply it early in the course of the disease. This treatment regimen has significantly reduced efficacy in patients with long-term resistance. It is crucial to use chemoimmunotherapy before chemotherapy resistance mechanisms develop. The earlier the regimen is implemented, the greater the chance is of synergy between chemotherapeutic agents and anti-GD2 antibodies. The synergistic effect of these components when administered early improves the rates of CR, and this may result in increased rates of several-year survival. This suggests the potential of using a method based on combining naxitamab with chemotherapy. In addition, an advantage is that the treatment can be administered in an outpatient setting and is well tolerated by patients [53,58].

### 5.3. Naxitamab and Nanofenretinide/Nanospermidine

Preclinical studies. Galassi et al. evaluated the efficacy of the combination of nanofenretinide (NF) with naxitamab and nanospermidine (NS) with naxitamab [61]. NF is a complexed synthetic retinoid that has anticancer activity while NS is one of the polyamines with cytotoxic effects in the form of nanomicelles [62,63]. SH-SY5Y (without amplification) and CHP-134 (with *MYCN* amplification) NB cells were treated with NF or NS at a concentration of 0.05 mg/mL in combination with a naxitamab and then tetrazolium salt test with 3-(4,5-dimethylthiazol-2-yl)-2,5-diphenyltetrazolium bromide (MTT) was conducted. The results showed that in the CHP-134 line, NF in combination with naxitamab reduced tumor cell viability to a greater extent than individually, while NS with naxitamab had no significant effect. The inhibition of cell confluence was proportional to the concentration of naxitamab. In the SH-SY5Y line, neither combination had a significant effect on tumor cell confluence. Nonetheless, it was shown that the combination of NS + naxitamab inhibited cell motility, which affects tumor invasion and metastasis. In addition, the study showed the increased fluorescence of cells treated with NS and NF, indicating an increase in the expression of GD-2 molecules and, thus, an increased intensity of the antibody-associated response. Considering that the efficacy of naxitamab depends on the expression of GD2 on cancer cells, this solution seems to have potential. The results of the study indicate that it is worth considering combining naxitamab with NF or NS to improve anti-GD2 treatment response and efficacy. However, in vivo clinical trials are necessary, where these results may still be improved due to the involvement of immune cells mediating ADCC and antibody-dependent phagocytosis (ADP) [61].

## 6. Combining Immunotherapies Based on CAR-T

An immunotherapy approach that has shown good results in the treatment of hematologic malignancies is chimeric antigen receptor T cell therapy (CAR-T) [64]. CAR-Ts are genetically modified lymphocytes that express chimeric antigen receptors (CARs). A CAR has two ends: the “recognition” and the “activation” end. The “recognition” end contains a recombinant fragment of the single-chain variable. The “activation” end results in T lymphocyte proliferation and the killing of target-bearing cells [65]. The most commonly studied targets for the treatment of NB are GD2, GPC-2, B7-H3, and L1-CAM [66]. Due to the suppressive tumor microenvironment (TME), the lack of specific antigens, and the reduced survival of CAR-Ts in the circulation and tumor site, the application of therapy in solid tumors has limited effects [67]. Compared with the outcomes of patients treated with CD19.CAR-T in acute lymphoblastic leukemia, response rates for GD2.CAR-T treatment in patients with NB are significantly worse [68]. Studies with CAR-Ts targeting GD2 in NB demonstrated safety but limited efficacy [69]. Combining the therapy with other methods to increase the effectiveness of CAR-T appears to be a promising approach.

### 6.1. CAR-T and Immune Checkpoint Inhibitors

The presence of CAR-T cells targeting NB may induce the programmed death receptor 1 (PD-1)/programmed death-ligand 1 (PD-L1) checkpoint signaling axis [70]. The PD-1/PD-L1 axis is one of the main immune checkpoints that suppress the antitumor immune response [71]. Positive PD-L1 expression is associated with reduced OS in patients with NB and higher levels of tumor markers [72]. One strategy to increase antitumor response is to combine CAR-T therapy with PD-1 blockade using checkpoint inhibitors such as nivolumab [70]. PD-1 blockade, by transforming the “cold” TME into an immunologically “hot” one, can increase the effectiveness of CAR-T cells [70]. However, for this strategy to be effective, PD-L1 must be present on NB cells, and according to studies, its expression varies in diagnostic biopsies [70].

*Clinical studies.* Heczey et al. conducted a phase 1 non-randomized study (NCT01822652) using CAR-T cells with PD-1 inhibition and lymphodepletion. The study lasted from August 2013 to December 2015. The main aim was to assess the clinical safety of GD2-CAR3 T cells in patients with R/R NB. Eleven patients from 4.1 to 23.6 years with a median age of 6.5 years with R/R NB were enrolled in three cohorts. One patient had a stage 3 pelvic tumor that was unresectable. Ten patients had stage 4 NB with the most common metastases involving bone and bone marrow. Four patients were enrolled in cohort 1: two received 1 × 10^7^ and the other two received 1 × 10^8^ GD2-CAR3 T cells intravenously. In cohort 2, four patients were enrolled: two received 1 × 10^8^ and the other two received 1.5 × 10^8^ GD2-CAR3 T cells intravenously. Cyclophosphamide (Cy) at 500 mg/m^2^/dose on days -4, -3, and -2 and fludarabine (Flu) at 30 mg/m^2^/dose on days -4 and -3 were additionally administered intravenously. In cohort 3, three patients were enrolled who intravenously received 1.5 × 10^8^ GD2-CAR3 T cells, and Cy/Flu and the PD-1 inhibitor pembrolizumab were intravenously administered on days -1 and 21 at 2 mg/kg/dose. The response to treatment was assessed at week 6 (day 42) following CAR-T cell infusion. Cy/Flu was shown to induce lymphodepletion, which increased interleukin-15 (IL-15) levels (*p* = 0.003) and CAR-T expansion (*p* = 0.03). PD-1 inhibition in cohort 3 did not increase CAR-T lymphocyte expansion and persistence further. In addition, the expansion of CD45, CD33, CD11b, and CD163 myeloid cells was observed in all patients. The treatment was well tolerated and safe, with no dose-limiting toxicities. Fever was present in all cohorts: in two patients in cohort 1, in one in cohort 2, and in two in cohort 3. Fever with neutropenia was observed in two patients: one in cohort 2 and one in cohort 3. A patient in cohort 1 had an episode of cytokine release syndrome (CRS), which only required prophylactic antibiotics and resolved spontaneously after 7 days. In cohort 1 (without Cy/Flu), hematologic complications—grades 3 and 4 leukopenia, neutropenia, lymphopenia, and thrombocytopenia—were observed less frequently compared to in cohorts 2 and 3 (*p* < 0.001). The Cy/Flu-receiving cohorts (2 and 3) showed higher levels of IL-15 during CAR-T cell infusion compared to cohort 1 (with Cy/Flu: mean 4.27 pg/mL, SD: 2.59; with Cy/Flu: mean 43.25 pg/mL, SD: 18.26; *p* = 0.003). Higher levels of circulating IL-15 correlated with the increased expansion of GD2-CAR3 T cells, as assessed via Spearman’s correlation (r = 0.796; *p* = 0.006). At the 6-week follow-up, six patients had PD while five had SD. The patient with stage 3 disease treated in cohort 3 after treatment showed a small residual pelvic mass that was resectable. The median survival of 506 days for all patients was reported. Disease progression and death after 32–506 days (median 230 days) occurred in all four patients in cohort 1. In cohorts with lymphodepletion (2 and 3), six of seven patients survived at >265 to >724 days. Two of these patients (in cohort 3) achieved CR [73].

Preclinical studies. Toews et al. conducted an in vitro study in which they examined whether the combination of CAR-T cell therapy with a checkpoint inhibitor (nivolumab) had the effect of counteracting the immune system’s escape mechanisms, thereby increasing anticancer effectiveness. Three NB cell lines differing in the level of surface density of the target L1CAM antigen were used in the study. The SK-N-BE(2) cell line was used, which was characterized by an average density of this antigen, while the SH-SY5Y and SK-N-AS lines expressed low levels of the antigen. Second-generation CAR-T cells were generated from CD8 + T-enriched CM cells, and a comparable CAR expression was ensured through immune selection for EGFRt. PD-L1 expression was significantly induced in all three cell lines when cocultured with T cells that expressed L1CAM-targeted CAR, in addition to control T cells. Before encountering CAR-T cells, these cells expressed low levels of PD-L1. The study also demonstrated that nivolumab-enhanced L1CAM-CAR-T cell cytotoxicity was based on significant PD-1/PD-L1 expression and, in turn, was influenced by the co-stimulatory domains in the CAR construct. Therefore, the presence of PD-1 on CAR-T cells and the presence of the PD-L1 ligand on NB cells are necessary for the checkpoint inhibitor to increase the therapeutic efficacy of CAR-T cells. Therefore, it was concluded that when choosing a therapy using CAR-T cells and PD-1 checkpoint inhibition, the level of PD-L1 expression on NB cells should not be used to determine whether this method of therapy should be used in a given patient. However, the level of PD-1/PD-L1 expression on CAR-T cells and the selection of a subset of T cells for the production of CAR-T cells will be important [70].

### 6.2. GD2-CAR-T and Bevacizumab

In the course of cancer, there is increased neoangiogenesis, induced mainly by the vascular endothelial growth factor (VEGF), which has an immunosuppressive effect on the TME and, thus, on the response to treatment. For this reason, one of the therapeutic options being considered is the use of antiangiogenic drugs that would support tumor infiltration by lymphocytes after inducing changes in the tumor vasculature. Hence, combining the effects of antiangiogenic drugs with CAR-T cell immunotherapy has been proposed as a potential therapeutic option [71].

Preclinical studies. Bocca et al. examined the anti-NB activity of GD-2 CAR-T cells combined with bevacizumab (BEV) in an orthotopic human NB xenograft model. Bevacizumab, which was used in the study, is a humanized monoclonal antibody against human VEGF-A approved for use in cancer therapy. Two weeks after the tumor was implanted in the mice, BEV or GD2-CAR-T cells or BEV and GD2-CAR-T cells were administered. Significant GD2-CAR-T cell activity was revealed only in combination with BEV. In this combination, GD2-CAR-T cells intensively infiltrated the tumor mass and produced interferon-γ (IFN-γ) there. IFN-γ and possibly other cytokines increased PD-L1 expression in NB cells. GD2-CAR-T cells that infiltrated the tumor expressed PD-L1; therefore, the antitumor efficacy of combining GD-2-CAR-T cells with BEV may be limited by the PD-1/PD-L1 axis. The results of this study justify the conduct of clinical trials in patients with NB, in which the combination of GD2-CAR-T cells with BEV will be tested [71].

## 7. Future Directions

Several of the therapies being developed for neuroblastoma treatment have not reached clinical trials yet. Future directions in preclinical trials are shown in Table 4.

Immunotherapy and photothermal therapy. In relation to the current limitations of methods that have already been used in the treatment of NB, new approaches are still in demand. Thereupon, treatment models that combine immunotherapy and stimulate the host response with targeted methods that inhibit molecular pathways crucial to tumor development are being explored [74]. A therapeutic model based on a combination of immunotherapy and photothermal therapy (PTT) appears to be promising. PTT is a relatively new strategy in cancer treatment, involving the use of heat from optical energy that is absorbed by light-absorbing agents present in the tumor [75]. In addition, it has been discovered that PTT, using inorganic nanocarriers, can potentiate the antitumor effect [76,77,78]. Recently, the focus of attention has been on the association of PTT with immunotherapy—immune adjuvants, checkpoint inhibitors, or adoptively transfected effector cells [75,79,80].

In view of the existing potential for such combination therapy, Sekhri et al. conducted a study on Prussian blue nanoparticle-based photothermal therapy (PBNP-PTT) to analyze the effect of thermal dose on the correlates of the immunogenicity of human NB cells in vitro. The effects of thermal doses on a cell line with *MYCN* amplification (LAN-1) and without *MYCN* amplification (SH-SY5Y) were compared. The PBNP-PTT dose was controlled by independently changing the concentration of Prussian blue nanoparticles (PBNP) in the cells and the near-infrared laser power. It was observed that thermal doses >5 log(∑CEM43) caused maximal immunogenic cell death (ICD) in SH-SY5Y cells and doses ≥10 log(∑CEM43) in LAN-1 cells, indicating that a higher thermal dose is required to produce ICD in LAN-1. The determinants of ICD were decreased intracellular ATP, increased extracellular HMGB1, and the increased expression of calreticulin on the cell surface. Also, in terms of the increased expression of CD80 and CD86 molecules, which are crucial for T cell function, SH-SY5Y cells required a lower thermal dose (≥5.3 log(∑CEM43)) than LAN-1 cells (≥11.3 log(∑CEM43)). In SH-SY5Y cells, both HLA-ABC and HLA-DR increased after PBNP-PTT, while in LAN-1 cells, only HLA-DR increased. Considering that the downregulation of HLA-ABC and HLA-DR is an important strategy to evade tumor cell immunity, the use of PBNP-PTT represents a strategy to recover immune surveillance in NB. Moreover, in response to thermal doses of PBNP-PTT, GD2 expression was significantly increased on the surface of SH-SY5Y cells, which provides important information regarding the potential of anti-GD2 immunotherapy-based combination therapy with PBNP-PTT for the treatment of NB without *MYCN* amplification. The results of this study indicate that PBNP-PTT in combination with immunotherapy may be an effective method in eradicating NB, albeit with much higher efficacy in tumors unrelated to *MYCN* amplification. It is possible, however, that combining this method with MYC-targeted drugs may provide results similar to the treatment of tumors without amplification [81].

There is a study described, which was conducted on mice, in which PBNP treated with a molecular adjuvant, CpG oligodeoxynucleotides (CpG-PBNP), were used in PTT in combination therapy with an anti-cytotoxic T lymphocyte-associated protein 4 (CTLA-4) checkpoint inhibitor. It has been shown that this therapy can be an effective solution to overcome the blockade of PTT’s abscopal effect caused by the prevention of effective T cell function by the immunosuppressive environment of the tumor interior. CpG-PBNP-PTT with anti-CTLA-4 in vivo caused complete tumor regression in mice. Moreover, mice treated with this method showed immunity and memory response against tumor cells. This study indicates that modified nanoparticles combined with immunotherapy provide options to combat the limitations of immunotherapy. These results could be the basis for continued clinical trials and eventually the use of this therapy to treat patients with NB [79].

Yes-Associated Protein knockdown. Yes-Associated Protein (YAP) is a transcriptional co-activator and major effector of the Hippo pathway, which regulates genes promoting organ growth, survival, and cell self-renewal [82,83,84]. Its overexpression has been found in various cancers, including NB with transcriptional activity correlated with an advanced grade and increased in relapsed tumors [85]. The activation of YAP signaling is engaged in promoting tumor cell proliferation, angiogenesis, therapy resistance, and metastasis [86]. The overexpression of YAP in NB is associated with negative outcomes and a poor prognosis [87,88,89]. Previous studies have shown that YAP inhibition suppresses tumor growth and cisplatin resistance in NB and may improve chemotherapy response [90,91,92]. The latest studies have shown that the increased expression of YAP also enhances resistance to anti-GD2 immunotherapy in HR-NB [93,94]. One study showed SK-N-AS shYAP cell line sensitization to γδ T cell killing with and without dinutuximab as a result of YAP knockdown. A panel of NB cell lines was evaluated and an inverse relationship in YAP and GD2 expression on the cell surface was noted [94]. Pilot results from a study with SK-N-AS or shYAP xenografts receiving an 18-day treatment with dinutuximab, γδ T cells and cyclophosphamide showed extended survival in mice [94]. Recently, Pilgrim et al. found that anti-GD2 immunotherapy resistance in NB is mediated by YAP through the downregulation of *ST8SIA1*, the gene encoding GD2 biosynthesis enzyme. Following the genetic inhibition of YAP, the increased expression of *ST8SIA1* was demonstrated in SK-N-AS NB cells, resulting in increased GD2 expression on the cell surface and increased sensitivity to γδ T cells and dinutuximab in vitro and in vivo. Combining immunotherapy with YAP knockdown is a promising therapeutic target for the treatment of NB, especially in cases of relapse [95]. Further research including preclinical and clinical trials is needed to develop new therapies focused on YAP and the Hippo signaling pathway, whose inhibition may improve outcomes in patients with tumors resistant to anti-GD2.

Oncolytic viruses and immune checkpoint inhibitors. NB is an immunologically “cold” tumor with reduced lymphocyte infiltration in the TME [96]. This results in a poor response to immune checkpoint inhibitors (ICIs). A promising approach is combination therapy using vaccines with oncolytic viruses (OVs), which enhances the anticancer immune response. OVs induce tumor inflammation and increase immune cell infiltration, which transforms the TME to immunologically “hot”, resulting in a better response to ICIs. Moreover, OVs are competent to selectively replicate and kill cancer cells [97,98,99,100,101,102,103]. Preclinical studies of NB treatment with OVs have shown tumoricidal effects [104,105,106,107,108]. In clinical trials with OVs, good tolerability and low toxicity were observed, but not all studies demonstrated CR or PR in the treatment [109,110,111,112]. The efficacy of OVs in combination with ICIs in the treatment of cancers such as melanoma, glioma, colorectal cancer, and non-small-cell lung cancer has been confirmed by clinical trials [113,114,115,116,117,118]. Hallenbeck et al. investigated the efficacy of immunovirotherapy in the treatment of NB. Seneca Valley Virus (SVV-001) was injected into a tumor in a syngeneic mouse model of NB, resistant to ICI therapy. This resulted in a reversal of anti-PD-1 resistance and increased efficacy. The combination of SVV-001 and anti-PD-1 enhanced the response rate 6-fold compared to SVV-001 monotherapy (*p* < 0.01) and 3–6-fold compared to ICI alone (*p* < 0.01) and improved the survival of mice [119]. Immunovirotherapy seems to be a promising approach for the treatment of high-risk and recurrent NB, with low toxicity for the patients. More research is needed.

Fostamatinib (R788) and anti-PDL-1 monoclonal antibodies. Fostamatinib (R788) is the first spleen tyrosine kinase (Syk) inhibitor approved for therapeutic use [120]. Its first global approval occurred in 2018 for the treatment of adult patients with chronic immune thrombocytopenia [121]. Rohila et al. investigated the therapeutic targeting of Syk with fostamatinib alone and in combination with anti-PDL1 mAb in NB using syngeneic cell lines with *MYCN* amplification and myeloid Syk KO mice. They demonstrated that Syk is a marker of macrophages associated with NB and its blockade in NB mice significantly impaired tumor growth. Furthermore, the combination of fostamatinib with anti-PDL1 mAb led to complete tumor regression and sustained antitumor immunity in mice with small tumors (50 mm^3^). The addition of radiation prolonged the survival (*p* < 0.001) of mice with large NB9464 tumors. The conversion of the TME and sensitization of NB tumors to anti-PDL-1 mAb via Syk inhibition using fostamatinib, with anti-PDL1 mAb and radiation, work synergistically and appear to be a promising strategy for NB treatment [122]. Further investigation in clinical trials is desired.

**Table 4:** Future directions in preclinical trials.

Method	Treatment Elements	Results	Comments	References
Photothermal therapy + immune checkpoint inhibitor	Prussian blue nanoparticle + CpG oligodeoxynucleotides Anti-CTLA-4	Complete tumor regression in mice	Mice developed immunity and memory response against cancer cells	[79]
YAP knockdown and dinutuximab	Genetic inhibition of YAP Dinutuximab, γδ T cells, and cyclophosphamide	Extended survival of mice	YAP inhibition increases sensitivity to dinutuximab	[94]
Oncolytic virus and immune checkpoint blockade	Seneca Valley Virus Anti-PD-1 mAb	Extended survival of mice	The combination increased the response rate compared to monotherapy	[100]
Spleen tyrosine kinase inhibitor and anti-PDL-1 monoclonal antibodies	Fostamatinib Anti-PDL1 mAb	Complete tumor regression in mice with small tumors (50 mm^3^)	Adding the radiation extended the survival of mice with large tumors	[122]

Disulfiram. A new candidate drug for the treatment of HR-NB is disulfiram, a drug used to treat chronic alcoholism. Beaudry et al. showed in preclinical studies that disulfiram at low nanomolar concentrations significantly delayed tumor progression in vivo. Disulfiram showed anticancer effects after 48 h of exposure to neuroblasts. Lysine acetyltransferase 2A (KAT2A), a protein whose levels decreased under the influence of the drug, was identified as a novel target of disulfiram [123]. To date, clinical trials have shown improved OS in adult patients with high-risk breast cancer and non-small-cell lung cancer treated with disulfiram and its active metabolite, sodium dithiocarb [124,125]. Moreover, it has been shown that continuous treatment with disulfiram was associated with a lower risk of cancer death compared to stopping the drug upon cancer diagnosis [126]. The advantage is that the long-term intake of disulfiram is safe and adverse effects are negligible. The inhibition of aldehyde dehydrogenase only occurs after alcohol consumption; therefore, its repurposing for children appears ideal. Disulfiram may demonstrate synergistic effects with other anticancer therapies. Combination treatment with disulfiram and immunotherapy seems to be a promising direction; therefore, further preclinical and clinical studies are needed [123].

## 8. Conclusions

Combination therapy, by targeting different mechanistic pathways, can achieve better response rates, as well as prevent the complications of disease and reduce drug toxicities. The combination of immunotherapy with other drugs improves its effects and clinical outcomes compared to single-drug therapy. Despite the improvement achieved via the introduction of immunotherapy in NB, patient survival rates are still not satisfying. The search for new therapeutic targets and combination treatment strategies for HR-NB is essential to increase survival rates in this cancer in the future.

## Figures and Tables

**Table 1:** Treatment of NB according to risk group.

Pretreatment Risk Group	Proportion of Patients	Current Treatment
Very low and low	55% [2]	Observation alone or surgical resection
Intermediate	9% [2]	Neoadjuvant chemotherapy, surgical resection
High	36% [2]	Intensive multimodality treatment including: (I) Induction: surgical resection, multiagent chemotherapy (II) Consolidation: autologous stem cell transplant, high-dose chemotherapy, radiotherapy (III) Maintenance: anti-GD2 immunotherapy, isotretinoin

The most frequently administered conventional cytotoxic chemotherapies include topotecan with either cyclophosphamide or temozolomide or irinotecan and temozolomide [14]. The North American model includes five cycles of chemotherapy. For cycles 1 and 2, the patient receives topotecan/cyclophosphamide; for cycle 3, the patient receives cisplatin/etoposide; for cycle 4, the patient receives vincristine/doxorubicin/cyclophosphamide; and for cycle 5, the patient receives cisplatin/etoposide [6]. The COJEC system, on the other hand, contains cisplatin [C], vincristine [O], carboplatin [J], etoposide [E], and cyclophosphamide [C], received every 21 or 10 days [13].

## Data Availability

Not applicable.
